# Experiencing Event Management During the Coronavirus Pandemic: A Public Sector Perspective

**DOI:** 10.3389/fspor.2021.814146

**Published:** 2022-01-10

**Authors:** Tim Coles, Giselle Garcia, Evelyn O'Malley, Cathy Turner

**Affiliations:** ^1^University of Exeter Business School, Streatham Court, Exeter, United Kingdom; ^2^London College of Music, University of West London, London, United Kingdom; ^3^Department of Drama, College of Humanities, University of Exeter, Exeter, United Kingdom

**Keywords:** events, coronavirus, local authority, performing arts, officer, United Kingdom, management, transformative

## Abstract

Events have played a significant role in the way in which the Coronavirus pandemic has been experienced and known around the world. Little is known though about how the pandemic has impacted on supporting, managing and governing events in municipal (i.e., local) authorities as key stakeholders, nor how events have featured in the opening-up of localities. This paper reports on empirical research with senior events officers for local authorities in the UK on these key knowledge gaps. Specifically, it examines events officers' unfolding experiences of the pandemic. The paper points to unpreparedness for a crisis of this scale and magnitude, and the roles of innovation, adaptation and co-production in the emergent response. It highlights the transformative nature of the pandemic through reconsiderations of the purpose of public sector involvement in events and, from a policy perspective, how relatively smaller-scale, more agile and lower-risk arts events and performances can figure in local recovery. Finally, while the effects on, and response of, the body corporate (the local authority) to crises is an obvious focus, it is important to recognise those of the individuals who manage the response and drive change.

## Introduction

Events have played a significant role in the way in which the Coronavirus pandemic has been experienced and known around the world. As potential vectors for the transmission of the virus, the continuation of some major sporting events in Spring 2020 caused controversy in the United Kingdom (UK, Conn, 2020) while the subsequent cancellation of events was an obvious manifestation of the new restrictions on personal mobility (Aspinall, 2021). A year later, events were used as a testbed for the efficacy of public health measures, and for assessing how safely and practicably to restart activities that used to be considered “normal” (Government, Her Majesty's, 2021a). Approaches of this nature demonstrate a new political calculus involving the balancing of public health with wider considerations. Judging the right time to restart events reduces transmission and saves lives, but it also limits the losses to, and strains on, events organisers and promoters, businesses in the extended supply chains, and the many livelihoods that depend on them [Culture, Media and Sport Select Committee, House of Commons (CMSSC), 2020; Digital, Culture, Media and Sport Committee, House of Commons (DCMSC), 2021a].

The experience of, and response to, the pandemic has been particular to each country [Organisation for Economic Co-operation and Development (OECD), 2020a]. Practise-based narratives in the UK have concentrated on the need to provide adequate support to the sector because of the significance of events of all types to economy, society and culture as well as everyday lives [Business Visits Events Partnership (BVEP), 2020; Organisation for Economic Co-operation and Development (OECD), 2020a; Digital, Culture, Media and Sport Committee, House of Commons (DCMSC), 2021b]. In articulating the scale of the pandemic, there has been a propensity to aggregate its effects on the sector as the rationale for leveraging government support [Business Visits Events Partnership (BVEP), 2020; Culture, Media and Sport Select Committee, House of Commons (CMSSC), 2020]. In so doing much of the diversity among events, festivals and spectacles is overlooked. Moreover, because of their size and relative contribution, there has been a closer focus on the largest events and spectacles in the recovery [Department of Culture, Media and Sport (DCMS), 2021a]. Much of the emphasis has been on private sector undertakings and their resilience to withstand the disruptive conditions [Department of Culture, Media and Sport (DCMS), 2021a; Digital, Culture, Media and Sport Committee, House of Commons (DCMSC), 2021a,b]. Conversely, the experience of the pandemic among public sector organisations involved in events has been largely overlooked, including the challenges they have faced reconciling their multiple roles and responsibilities.

As regulators and administrators of legislation, municipal authorities can act as the arbiters of where, when and how events take place in their sovereign territory (Maguire, 2019). In addition to acting variously as organisers, promoters and/or managers of events, public bodies may also own, operate and/or govern the spaces in which they take place and act as mediators among diverse stakeholders and their interests (Thomas and Wood, 2004; Wood, 2005; Devine and Devine, 2012, 2015; Maguire, 2019, 2021). Events are clearly important to the local economy and quality of life for citizens but these have to be balanced with the public health functions that local authorities, in the UK as in many countries, are statutorily obliged to enact, monitor and enforce [House of Lords (HOL), 2019]. Very little is known though about how the pandemic has impacted on the processes and practises for managing and organising events in or by municipal authorities. Nor has there been detailed attention to how events have featured in the gradual opening-up of visitor economies and the resumption of civic life in local communities.

The aim of this paper is to address these two significant knowledge gaps in the context of the UK. It reports on findings from research engaging those with greatest responsibility for managing and/or governing events for local authorities -hereafter “events officers” for convenience. As the embodiment of their municipalities for events, the paper examines how events officers experienced the pandemic, how they interpreted the role of events locally during 2020 and 2021, and how they viewed the future of events as their localities started to emerge from the pandemic. In so doing it makes three main contributions. First, it adds to our knowledge of how relatively small-scale events have featured in the response to the pandemic (Davies, 2020; Rowen, 2020). Second and connected, it points to the roles played by arts-related outdoor events in the emergence from lock-down. Small-scale performances and other outdoor art works have tended to fall between the cracks in the development of legislation or support during the pandemic [Culture, Media and Sport Select Committee, House of Commons (CMSSC), 2020], yet they have become a locus for innovative, creative ways of overcoming closure and social distancing measures (Braidwood, 2021). Third, it focuses on the people implementing events policies, their experiences and perceptions under extra-ordinarily challenging circumstances. Arts and culture are heavily reliant on public subsidy, the largest proportion from dwindling local authority budgets (Cooper, 2020). These have been under further strain from the pandemic [National Audit Office (NAO), 2021], thus placing unusual tensions on events officers. Paying attention to the ways in which local authority personnel perceive the transformative potential of the pandemic events therefore has significant implications for understanding the extent to which the sector may ultimately act as a force for beneficial change. We now turn to critically explore how events have featured in academic analysis of the pandemic.

## Literature Review

As the most disruptive peace-time event since the Second World War, the Coronavirus pandemic has attracted considerable attention from scholars of arts and culture, as well as tourism, leisure, and hospitality including events. Early contributions to the body of knowledge covering these intersecting activities and sectors, were clear in identifying, on the one hand, the nature and range of the potentially negative impacts of the pandemic and, on the other hand, the transformative potential of it for positive change (Hall et al., 2020; Sigala, 2020). As a public health crisis, the pandemic would prevent travel, tourism, cultural and leisure activities, including attending events in the ways they were known in the “old normal”. Following the pandemic, it was doubted whether production and consumption patterns would -and indeed should- revert to how they had been before: new behavioural conventions and expectations may induce a “new normal” (Ateljevic, 2020; Higgins-Desbiolles, 2020a). The pandemic could be a time to reset, to introduce new behaviours or systems for mediating and managing behaviours that had become inherently unsustainable (Benjamin et al., 2020; Ioannides and Gyimóthy, 2020).

Many of these early contributions took the form of “think pieces”, commentaries and provocations, often quite speculative in nature. This is understandable not least because a crisis of this scale or scope has not been encountered in recent memory. The absence of appropriate analogues and evidence bases to draw on did not aid substantiation and led to contestation, including some marked differences of opinion. Higgins-Desbiolles (2020b) identified sharp divergences in discourses between practitioner (i.e., industry) views and academic perspectives: in broad terms, the latter expressing more sympathy for the transformative potential of the pandemic and the desirability of a new normal; the former appearing to favour to the resumption of the old normal and sooner. Epistemologically, the dichotomy suggests a disconnect between the academy and those it seeks to study. It also points to a greater need for more work seeking to understand the issues from the perspective of those living and experiencing them. In a similar manner, Hall et al. (2020: 577) called for a more fully-considered response, cautioning “be careful of what you wish for”. Given the unprecedented nature and scale of pandemics as opposed to other forms of crisis and catastrophe, there may be unanticipated consequences for tourism, hospitality and leisure, including events. In other words, beneficial change would not necessarily be an outcome of the pandemic.

With respect to events scholarship, two features are apparent from this work. While events are included in general discussions about how the tourism sector (widely writ) may be impacted, they do not appear to have featured prominently in early research and discourse (Yang et al., 2021). Several commentaries and “think pieces” have speculated on the future for events. For example, Séraphin (2020) revisited existing propositions around the future of events (Getz, 2012) to conclude that virtual events will assume a greater role, events will remain a prominent feature of civilisation, but events will face -and have to be more resilient and adaptable to- more crises in the future. This echoes progress in theatre scholarship where there has been discussion of potential transformations in performance events, often focusing on digital modes of delivery and their significance in terms of aesthetics, reach and accessibility (Svich, 2020; Aebischer, 2021). In her discussion of festivals, Davies (2020: 188) observes that “smaller community festivals may be a more efficient method of limiting the number of attendees and cutting down costs for the festival hosts and attendees” while Olson (2021) identifies the pervasiveness of subversive practises during the pandemic, arguing for a research agenda on unauthorised and illegal events. Among the locations for unauthorised gatherings are public locations and venues, including “public parks, roadways, civic centres and convention centres owned by government” (Olson, 2021: 179). His work is notable for pointing to the need to think through the types of spaces used for social gatherings. Within the UK print media there has been a tendency to conflate discussions of indoor and outdoor events, yet an increase in outdoor theatre and venues has been noted (Braidwood, 2021; Caird, 2021; Goldborough, 2021; Kelly, 2021).

At the time of writing though, there have been few if any detailed examinations of the impacts of the pandemic on particular events -whether they have been or were about to be held (e.g., the Tokyo Olympics) or cancelled-, the implications for the destinations where they take place, and the consequences for the stakeholders involved in their organisation and operation. In notable but disparate exceptions, Maditinos et al. (2021) have explored runners' plans for which events to race in 2021. Adopting perspectives from organisational management, Séraphin and Jarraud (2021) have observed that, through ambidexterity, opportunities have been perceived and realised from the Lourdes pilgrimage despite the unpredictable context. Rowen's (2020) account of the *Burning Man* festival is unique for having considered transformation in the context of events. Drawing on responses to COVID and previous crises, he demonstrated “how values such as participation and civic responsibility may help people overcome shared conditions” (Rowen, 2020: 695). Finally, in studies of event management professionals (albeit from the private sector), Dillette and Sun-Ah Ponting (2021) employed Diffusion of Innovation theory to demonstrate that the pandemic has been a catalyst for creativity and innovation while in Portugal, 72% recognised the vital supporting role played by the public sector, especially in providing financial support to add to business resilience (Palrão et al., 2021).

In some respects, such limited attention should have been anticipated. With events cancelled, it is extremely awkward to conduct counter-factual research to assess the impact of something that has not taken place. Existing impact assessments of events, if they were undertaken before the pandemic, may offer some clues as to the nature and magnitude of any lost contributions. However, not all events have been the subject of rigorous impact assessments (Armbrecht and Andersson, 2016) and, moreover, the types of assessment and particular methods used can vary from event to event (Dwyer et al., 2000; Wood, 2005; Li and McCabe, 2012). Indeed, used in this way, existing impact assessments may understate the effects of a crisis or catastrophe, just as they underestimate the long-term effects under “normal” conditions (Jago et al., 2010). Nevertheless, given the potential vulnerability of events to crises and catastrophes and the (financial) risks this entails, it is surprising not to find more research on forecasting the potential effects of closure, restricted operations and possible development trajectories on stakeholders. Indeed, the lack of attention on the role public sector actors in event organisation during crises is a persistent problem (Devine and Devine, 2012). There is ample practical guidance on risk assessment, contingency planning, and operational management if a crisis or catastrophe happens during an event (cf. Bowdin et al., 2012), with some dedicated recommendations emerging from research on COVID-19 (Ludvigsen and Hayton, 2020; Chi et al., 2021). However, as Miles and Shipway (2020: 537) have observed, event management studies “could be better informed by disaster management and resilience studies”, furthermore contending that there has been a paucity of studies on managing crises and disasters for sport events in particular. This is amplified when compared with reviews of tourism studies, more widely specified (cf. Ritche and Jiang, 2019). Indeed, notable by its absence from their far-ranging review and research agenda, is consideration of the role of the public sector more generally and local authorities especially, in engineering resilience (e.g., contingency planning) or in mediating the response to crisis and catastrophe episodes. Local authorities are noted as potential beneficiaries of several pressing research imperatives (Miles and Shipway, 2020: 549) but overlooked as active agents in preparation for, mitigation of, or recovery from, crises.

In fact, local authorities have long been identified as major stakeholders in organising and operating events (Thomas and Wood, 2004; Wood, 2005; Maguire, 2019, 2021), and they play pivotal roles in co-ordinating responses to crises and catastrophes in visitor economies (Ritchie, 2009). For municipalities, participation in event planning, management or regulation can deliver a range of outcomes, not least economic benefits (Wood, 2005; Maguire, 2019). They may also be conceptualised as delivering a public good and justified on the basis of non-market benefits, including enriching local social and cultural life. As Andersson et al. (2020) demonstrate, there is a need to evaluate the value of events against risk in the public sector (just as in the private sector). Reward is jeopardised by a crisis or catastrophe, and extending their analysis, an episode like the pandemic clearly reframes the perception of risk and reward.

Although conducted in pre-pandemic times, analysis of this nature is helpful in two respects. First, it points to the importance of complex trade-offs in event management especially where local authorities may also act as investors in events. Decision-making about events is further complicated by the multiple and sometimes conflicting interests and responsibilities of local authorities, that have to be reconciled. For example, local authorities may have a statutory responsibility to protect public health or to ensure public events are safe. At the same time, local authorities provide events services, working on a fee-accruing basis with private sector operators, to support and/or operate events on public land or in public venues they own or operate. The salient point though is that, while the range and nature of local authority involvement in events management is relatively well-understood in pre-pandemic times (Thomas and Wood, 2004; Wood, 2005; Maguire, 2019, 2021), they have been largely overlooked during the pandemic, at a time when public sector intervention is keenly valorised by private sector operators (Palrão et al., 2021).

Second, a focus on decision-making points to a key lacuna in events management research on crises and catastrophes, including the pandemic, that has not been previously detected (cf. Miles and Shipway, 2020). It reminds us that it is easy to refer to the “public sector” or the “local authority” as the agency affecting, or being impacted by, change resulting from a crisis or catastrophe. Somewhat inadvertently, there is a tendency to conflate institution and individual, such that local authority and event professional (i.e., officer) are almost treated as synonymous with one another (Maguire, 2019). There is an assumed lack of difference between the institutional (the local authority) and the individual's position on, response to, or experience of, a crisis episode. Put another way, it is individuals who comprise or represent the institution (local authority), who embody and enact its values, and who are responsible for its day-to-day functioning in this domain. Yet, contemporary research extends the pre-pandemic feature of overlooking the lived experiences of local authority events officers (i.e., professionals). An omission of this nature is especially unfortunate in the context of the pandemic. After all, it is individuals who are immediately, directly and themselves, often corporeally confronted by crisis conditions both in their personal and professional lives. On behalf of the body corporate, they are pivotal to how crises and catastrophes are interpreted, understood and acted on, for instance in communications to internal and external stakeholders. From a functional perspective, they are especially well-placed to be able to articulate how (local) processes and practises are impacted by, and modified as a result of, episodes. Furthermore, they play key roles in determining how and when it is safe to resume events, and of what type, scale and scope. These are considerations in the remainder of the paper but it is important to observe that they contribute to filling major gaps in what we know about the pandemic has impacted on events and their management. The paper now explains the research conducted during 2021 to this end.

## Methods and Materials

Within tourism studies, there has been comparatively little attention on, and few possibilities to study, experiences among sector stakeholders and actors during a crisis or catastrophe as it happens (Ritche and Jiang, 2019). This is mainly because many such episodes are relatively short in duration (e.g., earthquakes, hurricanes, terrorist attacks). By virtue of its global scale and longer duration, COVID-19 has afforded new opportunities to investigate how protracted, larger-scale crises are experienced by stakeholders. As context to the study design, it is worth noting that local conditions have the potential to mediate differential impacts and experiences of the pandemic. Thus, before setting out in detail the methods used to collect and analyse the data, some salient background is sketched out briefly.

### Background

The pandemic has significantly disrupted the events sector in the UK (Culture, Media and Sport Select Committee, House of Commons (CMSSC), 2020; Digital, Culture, Media and Sport Committee, House of Commons (DCMSC), 2021a,b; Government, Her Majesty's, 2021a). In addition to the cancellation of many regular (annual) events, such as the festivals in Glastonbury and Hay-on-Wye, several landmark events that had been scheduled for 2020 and 2021, were reworked, postponed or cancelled. For example, in 2020 the city of Plymouth had organised events to celebrate the 400th anniversary of the sailing of the Mayflower to the New World while Coventry was due to take up its role as UK Capital of Culture in 2021. Estimates suggest that, prior to COVID-19, the sector generated £31.2 billion in visitor spend and 700,000 jobs across the UK [Business Visits Events Partnership (BVEP), 2020].

From a perspective of the governance of events in the UK, three features are particularly germane. First, although it may be vital to citizen quality of life, arts and culture are not mandatory (i.e., statutory) services that local authorities have to provide [House of Lords (HOL), 2019: 2]. Within England, local authority budgets for, and spending on, arts and culture have declined during the 2010s to around 2.3% or £3.5 billion in 2018/19 (Rex and Campbell, 2021). Second, as a mainstay of the visitor economy, not least for the promotion of both domestic and inbound tourism, events fall broadly within the portfolio of the Department for Digital, Culture, Media and Sport (DDCMS) in the central UK government at Westminster. Nevertheless, while DDCMS provides oversight, the day-to-day regulation, governance and administration of arts and culture (Durrer et al., 2019) as well as tourism and leisure, including events [Organisation for Economic Co-operation and Development (OECD), 2020b], is a devolved responsibility. This means that the governments in Scotland, Wales and Northern Ireland set their own agendas, priorities and arrangements independently from Westminster which acts as a proxy for England (i.e., the largest component of the UK by population). Third and connected, the same is also the case for (public) health (Paun et al., 2020), and during 2020 and 2021 arrangements relating to such issues as lockdowns, face mask wearing, social distancing and quarantining, varied subtly within and across parts of the UK.

### Research Design

The two knowledge gaps described in the introduction, provided the rationale for this study and constituted its over-arching research questions: how has the pandemic impacted on processes and practises for the organisation of events by municipal authorities, and how have events featured in the opening-up of visitor economies and the resumption of civic life? An important additional consideration was the extent to which there were variations around the UK based on devolved administration.

Qualitative research was used to address the questions and, due to the unprecedented circumstances, an exploratory approach was taken (Davies, 2011). As noted above, we aimed to engage with persons with the greatest responsibility for, and hence widest view of, events for their respective local government units in the UK. As a result, we would be able to access their perspectives on, and experiences of, events during the pandemic. Within England, this meant those responsible for events in district councils, unitary authorities or London boroughs and, in Wales, Scotland and Northern Ireland, their nearest equivalents. Although convenient, as a moniker “events officers” masks a range of institutional arrangements and professional roles among those participating in the research, an observation consistent with previous research (Thomas and Wood, 2004; Wood, 2005). This is not unique to the UK (Maguire, 2019, 2021) and, as the interviews for this study confirmed (see below), not all participants were exclusively or directly employed by local authorities. Some were employed outside the council apparatus, for instance in community interest companies or private limited companies established by local authorities to manage and service events (and frequently other) functions, with the municipality usually as the principal stakeholder and investor. Often these “arms-length” bodies also had remits for place promotion, (town/city) centre management, and attracting inward investment and visitors. In some case, they promoted or operated events, limiting the local authority's exposure to risk.

This study was conducted as part of a project funded by the Arts and Humanities Research Council (AHRC). Inter-disciplinary in nature spanning management and theatre studies, the project used a practise-based approach (involving the staging of outdoor arts events in a provincial English city), alongside interviews and surveys, to investigate the potential for, and role of, live events and performances in pandemic recovery. Smaller-scale arts events and performances were the focus because, although frequent and important before the pandemic, they had quickly fallen victim to the restrictions imposed following it [Culture, Media and Sport Select Committee, House of Commons (CMSSC), 2020]. Outdoor events were examined because -by virtue of lower transmission rates- they offered greater prospects for earlier, safer resumption (Braidwood, 2021; Goldborough, 2021). Moreover, they offered the potential for supporting an ecologically-responsible recovery, and even what theatre scholar Bharucha (2020, n.p.) terms “theatre cultures that mobilise ecological sanity”, avoiding “the hubris of the edifice complex”.

Data collection for this study was by semi-structured interviews conducted face-to-face *via* video link (MS Teams, zoom). Seven scripted questions were devised to facilitate cross-case comparison and provide a departure point for discussion. Table 1 explains the correspondence between the scripted questions in the protocol and the two research questions guiding this study. There were overlaps among the questions (and answers) as is common in interviewing but, broadly, five covered the impact of the pandemic and four explored the unfolding role of events in the immediate future. Functioning as a tacit quality assurance measure, the first questions also allowed us to establish the nature of the interviewee's organisational setting (*viz*. the local authority), its involvement with events in open-air public spaces, and the sorts of management and support for events, as a pre-pandemic baseline. A sub-text to Question Five was the uncertainty presented by evolving public health guidance and events guidance from government while Question Six offered space to explore the potentially the transformative nature of the pandemic for events. The final question may seem specific but should be read in the wider project context. As artists and performers deliver the main value propositions for events they are involved in (i.e., visitors are drawn to what they afford), this aimed to tease out how their working relationship evolved during the pandemic.

**Table 1:** Correspondence between scripted interview questions and over-arching study research questions.

**Scripted interview question**	**RQ(s) covered**
1. Tell me a bit more about the organisation, what it does, how it is involved with events in open-air public spaces?	1
2. How do you manage / support outdoor events in public spaces?	1
3. What sorts of outdoor events were you able to organise in public spaces from the start of the pandemic till now?	1
4. What sort of events are on the horizon / do you have in mind for the remainder of 2021?	2
5. What have been the main challenges in reopening live, outdoor events in public spaces?	1,2
6. Are there any examples of innovative, good and best practise among other local authorities and their approaches, that you look to or have informed your practise?	2
7. How have you worked with artists over the past year?	1,2

*Source: authors*.

*Study research questions (RQ)*.

*1. How has the pandemic impacted on processes and practises for the organisation of events by municipal authorities*.

*2. How have events featured in the opening-up of visitor economies and the resumption of civic life?*

Consistent with the exploratory approach, sampling aimed for maximum variation among local authorities and contexts. Interviews were sought in all parts of the UK; in both larger and smaller, more urban and rural municipalities; and with authorities including locations known for routine (i.e., annual) events as well as those with a recent or current involvement in major periodic events (i.e., celebrations of, “year of”). Frequently used in qualitative enquiry (Battaglia, 2008), a purposive strategy was employed. Here this comprised three elements. First, information about the project was distributed by mailing list maintained by professional communities of local authority events officers. This was notwithstanding the survey fatigue and low response rates sometimes observed during the pandemic (Patel et al., 2020). Second, our expert knowledge and that of professional contacts, was used to identify municipalities and officers where the issues may be highly resonant. Finally, a form of snowballing was used, with interviewees invited to recommend subsequent participants.

Context and timing were crucial: we attempted to investigate “live” issues as they were being experienced but this made sampling more difficult. Of all the devolved administrations, in February 2021 the UK government acting on behalf of England, the largest component of the UK, published the first “road map” to removing lockdown restrictions culminating in total release no earlier than 21 June (Government, Her Majesty's, 2021b). Comprising four steps in total, final design of the study (i.e., interview schedule and identifying participants) took place around Easter 2021 after early restrictions had been removed in Steps 1 and 2. Neither allowed organised entertainment events or large-scale public congregations. In parallel, an Events Research Programme was underway exploring “how events with larger crowd sizes could return without social distancing, while limiting the transmission of COVID-19 as much as practical” [Department of Culture, Media and Sport (DCMS), 2021b: 4], with a view to informing Step 3 (no earlier than 17 May). This allowed live events -indoor and outdoor- subject to capacity restrictions; the largest outdoor events of 10,000 or 25% of the seats, whichever was lower (Government, Her Majesty's, 2021b). Although delayed to 19 July, Step 4 marked the removal of all legal limits on social contact. Due to the disruption and additional work incurred by the delay some interviewees withdrew from the study while others had to postpone their interviews. Nevertheless, deferral offered the study the benefit of fuller, contemporaneous consideration of the issues as they were happening.

In qualitative research, there is sustained debate on sample size sufficiency (cf. Malterud et al., 2016; Hennink et al., 2017). Interviewing took place between May and August 2021 by which time common themes and recurrent points had emerged (Hennink et al., 2017). Fourteen interviews had been conducted of 36 mins average, the duration again a function of the availability of each interviewee. Recorded and fully transcribed, the data set comprised over 70,000 words. As all interviewees requested anonymity, thumbnail, contextual sketches of the municipalities are not supplied. In terms of data quality assurance, there was coverage across the UK, among different sizes of authority, and including locations hosting high profile events (e.g., annual festivals, spectacles) or programmes (e.g., cities of —). In other words, the sampling strategy achieved its purpose. Interviews were obtained from Wales, Scotland, and Northern Ireland as well as England, with non-representation from London the only gap. From the answers to the first interview question (Table 1) and cross-case comparison, there was no apparent bias to particular institutional settings or organisational arrangements, and the interviewees were qualified to speak in the capacity we had intended. Thematic Analysis was adopted to analyse the data and the patterns of meaning within it (Braun and Clarke, 2006). The transcripts were read, coded and reviewed, prior to the generation, confirmation and naming of themes, in a workflow akin to Nowell et al.'s (2017: 4) recommendation for good practise. As a commonly-used method both in organisational research (King and Brooks, 2018), the relative merits of applying this approach to foster rigour and trustworthiness in a range of contexts, are well documented (Nowell et al., 2017). Given the wide choice of techniques and approaches for analysing qualitative data, the choice of Thematic Analysis was appropriate here because the research was exploratory and intended to establish common interpretations and understandings from the data, rather than to build or test theory.

## Results

Four intersecting themes emerged relating how events officers experienced, and made sense of, the pandemic as it related to the territories and events over which they had jurisdiction. Two were connected by their interpretation of how the pandemic impacted on existing processes and policies relating to events in the recent past (i.e., research question one): the first, the current functions of event management, conveys views on existing approaches to support, management and governance and their appropriateness in the context of COVID-19; and the second deals explicitly with adaptation as a tactical response. Two further themes were rooted in the unfolding present and near future, exploring broader issues on the recovery of events and events in the recovery of civic life (i.e., research question two). These offer insights on the transformative nature of the pandemic, respectively dealing with the telos of event management and connecting events, space and the environment.

### Current Functions of Event Management

From the interviews, it is clear that the declaration of the pandemic and the challenges to event management practises, were unanticipated. The local authorities represented were not prepared for a crisis of this nature or magnitude. If suitable contingency plans were in place, they were not mentioned. One interviewee captured the difficulties of not having precedents or analogues to draw upon,

“We had all this historical knowledge about how people behaved and how things would work, and now it's like we're back to square one. Even if we've delivered an event plenty of times, we haven't delivered it post COVID and we don't know what impact that is going to have on crowd behaviour or audience behaviour.”

[Interview-6]

Interviewees variously tried to make sense of the situation and their ability to respond by reviewing the suitability of existing approaches to, and particular measures for, supporting the sector. Typical of this,

“we looked at what support could we lend to the festival sector, recognising the importance to XXXX of contributing £... million in economic impact and employing a huge number of people in XXXX, what we could do to support them and to deliver a different model that allowed them to transition from indoor venues to outdoor spaces and in terms of the financial support, not underrating ticket costs or risk or anything like that.”

[Interview-3]

Prior to the pandemic, the events officers reported that “their” organisations variously provided a “one stop shop”, enabling and facilitating events particularly piloting them through risk assessment and licencing, and in several cases, funding events, not just contributing to their organisation. Following the pandemic, core management and governance functions appear to have been provided by all, sometimes at reduced capacity (see below). For the majority though, it was no longer appropriate nor (financially) feasible to continue their funding of events, either at previous levels or at all (i.e., a temporary suspension). Resumption through 2020 and 2021 was a moot point. In general, changing public health guidance and government announcements introduced uncertainty and led to risk aversion. Events officers for Scottish local authorities noted the same broad effects but stressed the Scottish government's more cautious approach to releasing restrictions (cf. Castle, 2021).

While events were suspended, a minority of interviewees reported temporary changes to their roles and the configuration of the “events staff”. Some staff were redeployed to other (more) essential duties. As one noted,

“a lot of the time we were transferred to do other jobs.... A lot of stuff went on. I went to do Track-and-Trace, and so I was working there for a good 6 months.... I'm making the calls, asking people to isolate.”

[Interview-4]

Another events officer noted their local authority reduced events staffing by 57% during the pandemic,

“We went from a team of seven to three. We lost four staff during the pandemic as well, and they got new jobs.... so that's like three people working to try and, you know [.....] you can imagine three people trying to deliver all of XXXX's big outdoor events.”

[Interview-13]

Within England, the announcement in February 2021 of a “roadmap” to removing all restrictions (Government, Her Majesty's, 2021b), was an attempt to introduce certainty and enable planning for the resumption of regular (i.e., old normal) public services. An implicit assumption was that pre-pandemic arrangements were fit-for-purpose but this was far from universally the case for events. For several interviewees the return of the old *status quo* was unwelcome, with one complaining that “our events team have always been very risk adverse and very structured and very clinical” [Interview-5]. Experience of the pandemic had exposed many of the limitations in previous ways of working. For example, one events officer bemoaned the “silo mentality” of councils,

“And if one good thing comes from the pandemic, it is that those people, those groups and organisations have very much started back to work with each other. And I think the whole community spirit of work and joint working has come to the forefront.”

[Interview-14].

The disruption proved unsettling for most events officers. Cancellation of events and programmes troubled them not only because of the likely economic consequences but also because of the impacts on citizens and communities. Most events officers appeared to have forged strong professional networks with artists and performers. Over years of collaborating, a form of co-production had developed. Events officers and their teams got to know what artists and performers offered, the sorts of venues and facilities they needed, and the services they required from local authorities and other stakeholders. Events officers had commissioned artists and performers, and could approach them about their ideas for local events and programmes. Not surprisingly then, a sense of compassion permeated the interviews, both for those they had worked with directly as well as those in the wider sector,

“in the first couple of months, we were just, you know, supporting local businesses and events, trying to help them and push them towards the government funding opportunities available to all the people that were suddenly out of work.”

[Interview-4]

Summarily closing events appeared to threaten livelihoods. Rather than face lower incomes, many previously employed in events were used to the idea of the “gig economy” and took jobs in other sectors. The potential loss of experienced and capable performers and events workers was noted as a concern,

“One of the challenges we are facing is that people who were in the hospitality industry, they've gone through to other jobs and other work..... they've obviously realised that maybe being a delivery driver for our business is not as tough as trying to get gigs [and] will give us a more regular income.”

[Interview-1]

Reported by one interviewee as discussion point for LAEOG (the Local Authority Event Officers Group—a community of practice), the issue was widespread and created labour shortages and skills gaps that could hinder the resumption of events,

“Even in my autumn programme [in 2021], I'm struggling to get artists and struggling to get technicians because a lot of people have left the industry because they want more secure work.”

[Interview-5]

In other words, ensuring an adequate supply of labour had never been a major consideration before the pandemic. As one interviewee observed, although people were being lost from the events sector and may possibly not return, there was plenty of local talent that would embrace the opportunities they left. The challenge was how long it would take to fill the gaps and rebuild capacity. The inference was that it would be neither straightforward nor quick. Before the pandemic, regularisation seems to have been one approach to managing turbulence in the events “labour market”. In some respects echoing the previous observations about risk aversion, several events officers reported that repeatedly working with artists and performers was more straightforward “especially if someone's creating new work. They don't know what they're doing sometimes quite early on and what the impact of it is” [Interview-8].

### Adapting to COVID-19

In addition to recognising that different approaches were required to support, manage and govern events during the pandemic, the interviews reveal that considerable thought and creativity had been invested in addressing the question of what could be done “to allow festivals and events to continue safely?” [Interview-4].

Consistent with other studies (Dillette and Sun-Ah Ponting, 2021), there was strong evidence of innovation which takes a number of forms in the tourism-hospitality-leisure nexus (Hall and Williams, 2020). Institutional innovations -both in the form of general public health guidance and specific guidance for holding events- had to be interpreted and enacted. In some cases this led to cancellations; in others, advance ticket sales and bookings were adversely affected by the uncertainty created by intermittent changes to the guidance. As a result,

“we now see lots of people not commit to buying tickets or to confirm they're coming to something till really late in the day, whereas pre-COVID people would say things, you know, 6 months, a year in advance.”

[Interview-6]

In order to address the challenge of convincing audiences that events were safe, managerial innovations were introduced in the form of new administrative structures and systems. Perhaps most notable among these was a reorientation among “safety advisory groups”. Discussed further below, this involved a more direct and active involvement by public health officials in event organisation and management across the UK. There was some guarded positivity about the UK vaccination programme but uncertainty about vaccine passports and their practicability. Discussion among the community of practise reported by one events officer, suggested a trend for events administration was to go,

“down the route of having online toolkits and things like that, because what we need to do with the amount of work that's coming in..... is to try to have more and more online areas that people can go [for advice and to apply for licences].”

[Interview-14]

Capacity was a driver of managerial innovation. Pent-up demand among citizens was matched by private sector event organisers. COVID-19 had also catalysed “product” innovation in the form of both new and adapted events formats and programmes that needed to be understood and very carefully scrutinised before they were approved.

The need for product innovation was couched in the context of economic resilience and recovery. The possibilities and relative merits of using digital and hybrid spaces for delivery were frequently noted. While the benefits of “going digital” as a short-term response were identified, hybrid delivery could frustrate the economic purpose of staging an event,

“the whole point of it was to try and boost the retailers footfall but showing people things digitally isn't really isn't really going help the retailers.”

[Interview-5]

When local authorities eventually resumed organising events in 2020 and early 2021, they were routinely ticketed (to enable contact tracing to reduce transmission) but frequently free to enter (as a means of generating attendance although “no shows” were problematic). Streets, often in town centres were increasingly used for performance, installation and exhibition. As new solutions and COVID configurations, visual art was reported in outdoor installations and empty shop windows. Visual art appeared an effective way of re-engaging with publics because it attracted good “footfall” but without the costs and risks of drawing large crowds.

Particular performance-led outdoor events were also variously invoked as examples of both product and managerial innovations. A range of good and best practises for ensuring safety were identified, including *inter alia*: seated bubbles outdoors for audiences; increasing spaces between seating; “pop-up” performances in public spaces at specific times; and unannounced (walkabout) performances for those already in town centres spread virally through social media.

Initially at least, “COVID-safe” events appear to have been regarded as more costly and awkward to organise and manage. Among the various constraints that were repeatedly invoked were: insurance issues and other financial risks; extended lead times; and higher overheads, from requiring more space and/or greater administrative burdens. The withdrawal of sponsors and the temporary unavailability of the volunteer (public) workforce were further obstacles. One event officer encapsulated the issue,

“the big thing with events is people power, and it's having people on the ground..... And what I'm finding is that certainly in this city, the call on volunteers is absolutely massive.”

[Interview-14]

These issues were amplified for larger events, and one interviewee recalled a discussion with fellow events officers,

“no one felt overly confident as a local authority to deliver events in a COVID-safe way because no one as an event organiser has had that training. We got the [government] guidance, we know what we need to do. We understand the COVID-risk assessment and what needs to happen in terms of social distancing, hand-sanitising, that sort of thing. But in terms of practically delivering a large 4,000-person festival, we don't have that expertise.”

[Interview-10]

Smaller, outdoor events and formats were widely discussed. One pragmatic way of solving the expertise gap noted above was “rather than get people into to one big event to sort of have multiple events with three or four thousand rather than sort of have an eight twelve thousand that day” [Interview-14]. Of course, this view speaks to spreading risk by creative thinking. Paramount is that smaller events carry lower potential risk and rates of virus transmission. In addition, they are less expensive to organise and stage; they attract lower (financial) risk; and they are more agile and responsive to changing conditions. Furthermore, they put less pressure on other public services, especially the emergency services. In one city, larger events were deemed impossible because there was no “medical cover for events at the moment because the hospitals are still quite strained” [Interview-1].

### The Telos of Event Management

At this point it is worth observing that the telos of events management -or the questioning of the purpose(s) or objective(s) of what is done- has featured already. In their self-dialogues, event officers used the past (the “old normal”) to understand their experiences of the present. Besides their economic rationale, the social, moral and community-related purposes of events in the new operating conditions, were part of their considerations.

Teleological reflections were inherent to how events officers made sense of the future and their roles in shaping it. Capturing the essence, one interviewee reflected, “I think it's also made us take stock a little as well in terms of how we're going to deliver events in the future” [Interview-1]. Prior to the pandemic, the administration and management of events had become so routine that events officers had almost taken-for-granted the need for, and reasons why, events take place in their areas. The same interviewee observed that the size, scope and orientation of events were being reviewed, noting that an arts-based project was in prospect,

“a community event where they're going to create structures that people, the local community can help build.... we're going to have to look at more things like those or like, like trails.... rather than the big [XXXX] events, at least in the short term.”

[Interview-1]

An undercurrent among the interviewees was the appropriate scale and scope for events to engage people in local communities. No definitive position was forthcoming, perhaps not surprisingly. Local context mattered. Ultimately, scale and scope appeared to relate to what was perceived as safe. More than ever, the purpose of an event was to bring people together for benign, enjoyable experiences. As one interviewee put it, local authorities

“all have the same inherent aim, which is to make sure that an event happens safely, securely, and that people can have a good time and go home in the same condition that they went in.”

[Interview-6]

In this context, the changing composition, role and contribution of “safety advisory groups” was invoked by almost all events officers. Reported as discussed in the professional community of practise for events officers, the “big change.... across the country.... [had been] that public health now sit on safety groups which never existed” [Interview-1].

Convened by local authorities to ensure public safety at events, somewhat curiously before the pandemic they had not been attended by public health directors [or representatives]. As public health and event guidelines had been developed in response to the pandemic, their attendance and input into decision-making had become crucial. Capturing this sentiment, it was vital to,

“have access to clinicians who are able to go through their plans with them and give them kind of approval that what they're doing is safe or to offer them advice and to offer comfort that their plans are robust and in place.”

[Interview-3]

Although “advisory” in name, one interviewee noted that they had become far from this in practise and “advice” was, in practice, binding.

In many respects then, the pandemic had forced events officers to revert to the core purpose of events in thinking about the future. From the range of views, it appeared to be a chance to strip away some of the layers of complexity that had been added to event management and governance over time. In different ways, for the majority of events officers, the pandemic was a time when they could think about the social mission of the local authority and how this was best reflected through events and programming. Consistent with the notion of “building back better” from the “old normal” to a “new normal” (Ateljevic, 2020; Ioannides and Gyimóthy, 2020), the pandemic had been a time to reconnect and re-engage with local communities, and in two exceptional cases, a chance to develop new strategies. In the most progressive approach, co-design was being used to encourage residents of “all ages and all groups to kind of work with us” and the outcome of the exchanges would be a,

“framework so [arts and culture] organisations can take on board what co-design is, [in] how they with citizens in the city.”

[Interview-5]

Greater social inclusivity was evident in articulations of the future to reflect emergent local priorities and emphases, for instance in the representation of particular groups, especially minorities, in local communities. One event officer reported using the local authority's commitment to tackling the climate crisis and Net Zero emissions to reorientate their approach to “deliver more sustainable projects and how we can be a more sustainable and environmentally-friendly city” [Interview-12]. Another noted that one of “the council's main strategies is healthy living. So that's why they've gone down this route of offering the space for free” [Interview-4]. The local authority had waived booking fees on its sites in the short term, in order to encourage events as a vehicle for encouraging greater physical activity.

In other local authority areas, events officers reported efforts to align future events and programming more closely in support of placemaking and the representation of local geographies, cultures, histories and identities. One events officer captured this sentiment, arguing that the local events programme needed to project the image of “a global city now. We see ourselves as that, you know. So that's the direction we are taking” [Interview−13]. Identity-building was a continuing process which the pandemic had interrupted. Future events would play an important role in reiterating and reinforcing key messages to local and external stakeholders “because how would you know about XXXX or the history of XXXX or that kind of thing, since that event [and what it had to convey]....?” [Interview-1]

Finally, the idea of adopting a “hyper-local” orientation was evident to varying degrees because, “people, you know, by necessity [were] being forced to be very local in the lockdowns...” and they were “trying to really discover the neighbourhoods in a new way and on a new level of detail [Interview-8]. In one exceptional instance, this had progressed to thinking about,”

“how do we animate these local neighbourhoods, these local high streets. And because that's where people feel comfortable now, that's where they feel safer. That's what they are..... They haven't gone into the central town.”

[Interview-8]

### Events, Space, and Environment

The pandemic also encouraged significant thinking about the types of spaces and environments that support outdoor events in local municipalities, and their purposes. Prior to it, event officers and teams got to know the sorts of venues where particular types of events could take place locally, and which were routinely used for particular purposes. As one put it, “we've got a number of event spaces that we tend to use on a regular basis” [Interview-4], while another was,

“in a position where we can kind of talk from experience on what works and different areas, you know, whether you want it a certain type of space that benefits from existing footfall.”

[Interview-2]

The new conditions forced not only a reappraisal of the suitability of existing spaces but also a more creative approach to identifying alternative venues. On the former, several examples were offered of events and performances that had been adapted to conform with revised space and social distancing requirements, with some shrewd estimates as to the revised capacity of venues, as this rough-and-ready calculation indicates,

“a space that used to hold fifteen thousand people with social distancing will probably only hold five [thousand].”

[Interview-6]

In the case of the latter, there were several cases reported of events officers being challenged by artists and performers to come-up with novel, altogether different or original solutions. One events officer in particular explained that, somewhat surprisingly, artists and performers wanted to use outdoor spaces where there was not established footfall. Within the team, the disruptive nature of the pandemic seemed to have fostered a more adventurous and open-minded approach to considering alternatives. Although it meant going out of their “comfort zone”, they accepted the challenge because, as it was put: “something's difficult, but they're worth doing because the event benefits from it” [sic]. The same interviewee went on to note that the consideration and subsequent use of different locations created “event space in the city centre [that] was a huge sort of enabling factor for event organisers to seek to hold things in the city centre” [Interview-2].

A “can-do”, solutions-oriented approach was evident among all interviews to varying degrees, with the level of response a function of the resources available for public sector events management and local contexts, including size and configuration of urban settings. From the examples cited by events officers (as public servants), it became clear that considerable thought had been given to in how best to use public space for a public purpose. More potential spaces for outdoor events and performances had been identified than ever before, often with important potential benefits. Off-centre locations, industrial estates, car parks allowed events teams to “spread some of the impact to the further around the city, not just concentrated in the city centre” [Interview-3]. Rethinking spaces like parks, gardens, “green corridors” and so on, that had previously been overlooked or seldom used, raised some doubts about their prior under-utilisation. There were suggestions that, prior to the pandemic, some outdoor public spaces had been perceived as more difficult to manage for events. One common issue was containment in the sense of ticketing and controlling access to spaces, like parks and gardens, with permeable boundaries. In reassessing their suitability, issues that were previously considered possible limitations, like access at multiple entry points, offered potential advantages for managing post-lockdown events.

Within the interviews, the merits of several types of outdoor spaces were surfaced. In terms of common denominators, those owned or managed by the local authority appear to have offered lower risk of transmission in open air environments; greater accessibility (i.e., geographically and through transport systems, rather than relating to disability); and lower costs to potential event organisers (where the local authority did not act in this role). Challenges included the potential interruptions (i.e., to public transport, road closures) to the city and its people, as well as the need for new dialogues with stakeholders inside the local authority (e.g., the highways and environmental health departments) and external to it, including public transport providers, the police and emergency services. Other difficulties using green spaces (i.e., parks and gardens) included restrictive covenants placed on spaces, including: use only at certain times of the year; limits to the total number of days for use; and even protection orders preventing the use of particular spaces. Finally, some drawbacks in using public open space were reported. A dilemma appears to have been that, while open air settings may minimise the risk of transmission, open access makes monitoring of transmission and *post-hoc* contact tracing more difficult. To overcome this, several interviewees noted the use of large marquees and other structures in green spaces to provide a sort of “indoors-outside”: sufficiently large to allow air flow and social distancing; controllable from a public health perspective; and located in public space to minimise contact between attendees and residents.

## Discussion

What emerges from the interviews is a picture of the pandemic affecting practises and processes related to local events in complex and different ways, some of which could not have been anticipated from the existing body of knowledge. Perhaps most notably, this included reflection on the telos of the events they are involved in as a public sector actor, the event spaces used in their municipalities, and how events could be employed in, and contribute in a positive manner to, recovery from the pandemic. Despite varying governance arrangements during the pandemic, in this study there was insufficient evidence of contexts and conditions in different parts of the UK mediating detectable differences in experience in ways comparable to other studies (Dillette and Sun-Ah Ponting, 2021).

Events have featured in the reopening of visitor economies and the resumption of civic life, but evidence presented here suggests that this was perhaps not as quickly or as efficiently as may have been desired. One key assumption in “tourism scholarship” on the pandemic is that government support and intervention is necessary to develop both resilience and appropriate sector responses (Collins-Kreiner and Ram, 2020; Ioannides and Gyimóthy, 2020; Fang et al., 2021). Government would argue -especially at the national level for the UK- that it had increased its support -both in scale and scope- for businesses and organisations in the tourism-hospitality-leisure nexus, and for events as part of this [Department of Culture, Media and Sport (DCMS), 2021a]. In this study though, there was some, albeit limited evidence of a downsizing of local authority events functions and, more importantly, loss of capacity, knowledge and experience as events workers moved into other sectors. While this observation triangulates with other experiences reported in practise [Culture, Media and Sport Select Committee, House of Commons (CMSSC), 2020; Department of Culture, Media and Sport (DCMS), 2021b: 30], it suggests that early interventions and support were in some cases insufficient and too slow for events when compared to other elements of the tourism-hospitality-leisure nexus. It also hints that the nature and operation of live events was not fully understood in government [Department of Culture, Media and Sport (DCMS), 2021b: 30], and echoes the concern that exit strategies for tourism from crises are not always evidence-based (Collins-Kreiner and Ram, 2020).

Further policy development corroborates this finding. The first measures were not always effective in supporting event workers -including artists and performers- to protect their incomes and livelihoods. As an initial parliamentary enquiry revealed [Culture, Media and Sport Select Committee, House of Commons (CMSSC), 2020: 53], this was because they misjudged the preponderance of the self-employed and the structure of employment and remuneration (i.e., portfolio careers, freelancing and the gig economy) in the sector. Early support from the Arts Council focused on artistic teams core to “national portfolio” organisations, many artists and companies were not eligible for funding, and freelancers were precariously placed [Centre for Cultural Value (CVC), 2020]. Oversights of this nature compounded the problem that many artists and performers were poorly paid even before the pandemic (Jones, 2020). Indeed, in 2020, the uninsured risk of cancellation prevented the early return of many events [Culture, Media and Sport Select Committee, House of Commons (CMSSC), 2020: 44], and led to calls for a UK government underwriting of events during the pandemic [Department of Culture, Media and Sport (DCMS), 2021b: 39]. A scheme of this nature finally started in September 2021 (Government, Her Majesty's, 2021c) after the Tourism Recovery Plan had been published (June, 2021). Yet, as if to emphasise the indifference for smaller events as potential contributors to the recovery, announcement of the scheme stressed it was intended for “live music events, festivals, sports events, trade shows and business events” and covered those costs incurred before and by cancellation (Government, Her Majesty's, 2021c). It did not cover lower demand for tickets or reductions in capacity, and closer inspection raised questions of whether it represented a feasible financial option, especially for small events [Thomas and Smith, 2021; Department of Culture, Media and Sport (DCMS), 2021a]. Larger events, festivals and spectacles -especially related to popular culture and sport- deliver volume and value to the visitor economy, both quickly and conspicuously, and they symbolise the apparent return of “normality” [Department of Culture, Media and Sport (DCMS), 2021a].

There are three implications from this research for future policy and strategy if events are to be a force for good in the recovery from sustained, larger scale crises in the future. Paramount among these is the role for smaller, especially arts-related events in outdoor spaces. Findings from this research resonate with, and provide empirical substantiation of, Davies's (2020) and Olson's (2021) views, respectively, on the role of small and alternative public spaces for future events. With appropriate public health measures in place, smaller, outdoor events have the capacity to return citizens sooner than larger events to public spaces; to sociable, congregative behaviours; and to build morale and community spirit when it is tested most (cf. Rowen, 2020). Events of this nature are relatively agile and straightforward to set-up, more so where they benefit from established social networks, for instance among event workers in the gig economy, or the prior collaborations between artists, performers and events officers over time. As the experiences relating to Safety Advisory Groups attest, new public health guidelines can be incorporated into practise swiftly. Although there is undoubtedly a significant role for larger events in recovery, these are longer and more complex to organise and, even with insurance, the risk to, and vulnerability of, local visitor economies, business and citizens is never entirely eliminated.

Second, one legacy of the pandemic appears to have been to challenge the way in which events are thought about and managed in local government. While the “transformative” nature of the pandemic may connote grand aspirations (Ateljevic, 2020; Benjamin et al., 2020; Hall et al., 2020), interpreting the word in this way obscures its basic meaning, which relates to notable change. This is relative: set in context, even apparently modest-appearing actions can lead to marked difference. With orthodox views emerging on the organisation of events, increased awareness of the types of spaces that are available in the municipality adds to the range of amenities and facilities a local authority can offer post-pandemic. Teleological reflections on events and programming stress the importance of returning to the basics of safe, enjoyable events, later augmented by more of an emphasis on the publics that events officers serve. Prevailing policy in the UK may stress the value of events for economic recovery [Department of Culture, Media and Sport (DCMS), 2021a]. As vital as this may be, akin to other work (Rowen, 2020), this research also points to the role for interventions and support in maintaining and rejuvenating social relations. A key finding to inform future policy is that where the essential human infrastructure supporting events is disrupted, the ability to respond expeditiously and with agility is diminished. Within this research there was a keen interest of events officers in people, for instance the communities for which events were intended, or the artists, performers and workers who co-design, -organise and -deliver events. Put another way, economic development is an outcome but it is people that produce it.

Finally then, if events are vital to recovery, there is a need to focus support on retaining, training and upskilling local authority events officers as agents of change. While the prevailing approach may be to study the effects of crises on events at the level of the local authority, this research points to differences in the values and positions taken by the body corporate compared with those tasked with supporting, managing and regulating events on a day-to-day basis. Local authority spending on culture and the arts have been declining since the global financial crisis (Rex and Campbell, 2021), and budgets have been under further pressure during the pandemic (National Audit Office (NAO), 2021). Set against this backdrop, their personal dialogues and reflections suggest that -through attributes of understanding, empathy, compassion, creativity, innovation and problem-solving- their perceptions, assessments and decisions are pivotal to translating conditions and making change happen.

## Conclusion

This paper has explored how events officers experienced the pandemic as part of the public sector response in the UK to COVID-19 during 2020 and 2021. The practises and processes for supporting, managing and governing events within local authorities, were subject to some notable and unanticipated changes. This study has surfaced the role and value of smaller-scale, outdoor arts-focused events and performances in the local response to, and recovery from, the Coronavirus pandemic. Processes of adaptation and innovation involving events officers, artists and performers, enabled events to take place in challenging conditions. One of the most unexpected findings, at least from a policy perspective, has been the role that smaller-scale events and performances -especially arts-related and outdoors- can play as a force for good in recovery at the local level. While their contribution to economic revitalisation may be modest (and unattractive to some policy-makers as a result), they can play a notable -and not to be ignored- roles in reviving social and cultural life in local communities.

The potentially transformative nature of the pandemic on the tourism-hospitality-leisure nexus has been widely debated, although perhaps not as extensively with respect to events or arts and culture, specifically. The emergence of the teleological dimension in this study represents an important empirical corroboration of earlier academic conjecture and advocacy that the pandemic has had a transformative effect on events. However, it also points to limitations among existing theorisations of the transformative potential of the pandemic which overlook the role of individual event officers as the stimulus for, and the embodiment of, the public sector which plays such a key role in event organisation. In our view, the emergence from reflection and self-dialogue of a greater emphasis on the social and cultural functions of events for communities and citizens is a welcome reorientation to public sector events management. Of itself, it is transformative, a form of “building back better” if it results in a “new normal.”

The operative word is “if”. Replication studies are desirable to corroborate the main findings and policy recommendations described here. Further international research to offer comparative perspectives and experiences would be welcome, too. Most importantly though, the data collection for this study was completed in August 2021, broadly at a point when the process of “building back” started in earnest across the UK, following the easing of restrictions in England (Government, Her Majesty's, 2021b). For some, this may be regarded as a limitation of the study: possibly that is premature; even that the themes emerging here are temporally-contingent. In the case of the latter, it is almost axiomatic to question whether, were the work repeated in 2022 or 2023 (if the pandemic is continuing then), the same themes and precise emphases may emerge. This is the inherent challenge associated with the nature of qualitative enquiry: if this work were conducted later, the interviews' views may be different, conditioned as they may be by changing contexts and expectations. However, we would contend that this study is far from premature. While the Delta and Omicron variants have emerged in, and spread through, the UK since the research was conducted, the UK government, in particular, has been determined to continue along a path towards reopening society and economy. Although the devolved governments across the UK have responded to both variants with measures they believe are reponsible and commensurate, at the time of writing they have not required the closing down of events or venues. Put another way, they have tacitly enabled plans, positions and ideas articulated in this paper to continue and thereby to provide the basis for the ongoing recovery. Be this as it may, an obvious priority for future research is to return to the topic, preferably with the same interviewees to investigate the extent to which the telos of events has actually changed and transformed local (public sector) event management (or whether new variants and developments have prevented or altered this). In other words, it is necessary to understand whether events' officers reflections and aspirations have amounted to demonstrable changes over time in policy and practise towards supporting, managing and governing events at the local level. An implicit shortcoming in some of the work on the transformative nature of the pandemic for the tourism-hospitality-leisure nexus is that it is unspecific about when, for how long and in what nature transformation will take place. Equally, it is equivocal about who is responsible for delivering transformation. Events cannot be a force for good, and nor will the pandemic have the transformative nature its advocates intend, if there are not committed, passionate and capable people to make change happen.

## Data Availability Statement

The datasets presented in this article are not readily available because interviews were given on the strict understanding of anonymity. Requests to access the datasets should be directed to outsidethebox@exeter.ac.uk.

## Ethics Statement

The studies involving human participants were reviewed and approved by University of Exeter College of Humanities Ethics Committee. The patients/participants provided their written informed consent to participate in this study.

## Author Contributions

All authors listed have made a substantial, direct, and intellectual contribution to the work, and approved it for publication.

## Funding

This study was conducted as part of a project funded by the Arts and Humanities Research Council (AHRC), a part of United Kingdom Research and Innovation (UKRI) entitled Outside the box: open-air performance as a pandemic response (Award number: AH/V015230/1; https://openairperformance.com/).

## Conflict of Interest

The authors declare that the research was conducted in the absence of any commercial or financial relationships that could be construed as a potential conflict of interest.

## Publisher's Note

All claims expressed in this article are solely those of the authors and do not necessarily represent those of their affiliated organizations, or those of the publisher, the editors and the reviewers. Any product that may be evaluated in this article, or claim that may be made by its manufacturer, is not guaranteed or endorsed by the publisher.
